# Relation between cardiac dimensions and peak oxygen uptake

**DOI:** 10.1186/1532-429X-12-8

**Published:** 2010-02-01

**Authors:** K Steding, H Engblom, T Buhre, M Carlsson, H Mosén, B Wohlfart, H Arheden

**Affiliations:** 1 Department of Clinical Physiology, Lund University Hospital, Lund University, Lund, Sweden; 2 Department of Sport Sciences, Malmö University, Malmö, Sweden

## Abstract

**Background:**

Long term endurance training is known to increase peak oxygen uptake () and induce morphological changes of the heart such as increased left ventricular mass (LVM). However, the relationship between  and the total heart volume (THV), considering both the left and right ventricular dimensions in both males and females, is not completely described. Therefore, the aim of this study was to test the hypothesis that THV is an independent predictor of  and to determine if the left and right ventricles enlarge in the same order of magnitude in males and females with a presumed wide range of THV.

**Methods and Results:**

The study population consisted of 131 subjects of whom 71 were athletes (30 female) and 60 healthy controls (20 female). All subjects underwent cardiovascular MR and maximal incremental exercise test. Total heart volume, LVM and left- and right ventricular end-diastolic volumes (LVEDV, RVEDV) were calculated from short-axis images.  was significantly correlated to THV, LVM, LVEDV and RVEDV in both males and females. Multivariable analysis showed that THV was a strong, independent predictor of  (R^2 ^= 0.74, p < 0.001). As LVEDV increased, RVEDV increased in the same order of magnitude in both males and females (R^2 ^= 0.87, p < 0.001).

**Conclusion:**

Total heart volume is a strong, independent predictor of maximal work capacity for both males and females. Long term endurance training is associated with a physiologically enlarged heart with a balance between the left and right ventricular dimensions in both genders.

## Introduction

Long term endurance training is well known to increase peak oxygen uptake () and to induce changes in left ventricular dimensions and morphology [1-9]. However, it is not completely understood how  and cardiac dimensions are related and how endurance training affect the right ventricle and the total heart volume (THV). Furthermore, previous studies of the athlete's heart have mainly focused on male athletes and few studies include females. Thus, the gender aspects of the training effects on cardiac dimensions needs to be further explored.

Previously, chest x-ray has been used to determine the THV [10-13]. Two-dimensional x-ray has limited ability to describe the three-dimensional properties of the heart. Hence, in order to get a better understanding of the relationship between the THV and , a more accurate imaging modality is required. Cardiovascular magnetic resonance (CMR) is a completely three-dimensional imaging technique and is currently considered the gold standard for assessing cardiac function and dimensions [14]. Using this technique, it has recently been shown that left and right ventricles have different pumping mechanics [15]. It is therefore also of interest to explore if the relationship between the left and right ventricle is different in healthy controls compared to athletes.

Therefore, the aims of this study were to a) test the hypothesis that THV is an independent predictor of  in both males and females; b) to determine if the athlete's heart is a physiologically balanced heart where the left and right ventricular dimensions are enlarged in the same order of magnitude in both males and females; c) to examine gender differences in THV as well as LV and RV dimensions in athletes and controls.

## Materials and methods

### Study population and design

This study was approved by the local ethics committee and all participants signed a written form acknowledging informed consent.

Seventy-one athletes, 23 handball players (12 female), 30 soccer players (12 female), 18 triathletes (6 female), and 60 healthy control subjects (20 female) underwent CMR and maximal incremental exercise test on ergometer cycle with gas analysis. The soccer players and handball players were recruited from local elite teams competing at the highest national level. The triathletes had all been competing at national level for at least three years and trained for at least 10 hours/week.

None of the participants had a history of cardiovascular disease. All of the test subjects were non-smokers and did not use any medications with known cardiovascular effects.

Training intensity and frequency for handball players and soccer players was obtained from the responsible coach. Triathletes filled out a standardized questionnaire regarding training intensity and frequency. Healthy control subjects answered a questionnaire regarding their profession, leisure time and physical activity.

Both healthy controls and athletes were asked not to participate in any vigorous exercise 48 hours prior to the test, to be awake at least two hours before the exercise test, avoid heavy meals one hour before the test and not drink coffee, tea or eat chocolate two hours before the test. CMR and exercise test was performed the same day, always starting with the CMR at rest.

### Cardiac Magnetic Resonance (CMR)

A 1.5-T scanner (Philips Intera CV, Philips, Best, The Netherlands) with a cardiac synergy coil was used to scan all subjects in supine position. Images of the heart were acquired using a steady-state free precession MR sequence with retrospective ECG triggering (repetition time 2.8 ms, echo time 1.4 ms, flip angle 60°, spatial resolution of 1.4 × 1.4, temporal resolution typically 30 ms and slice thickness 8 mm with no slice gap). After defining the long axis orientation of the heart, short-axis images covering the entire heart from the base of the atria to the apex of the ventricles were obtained.

#### Volumetric measurements

**Figure 1:**
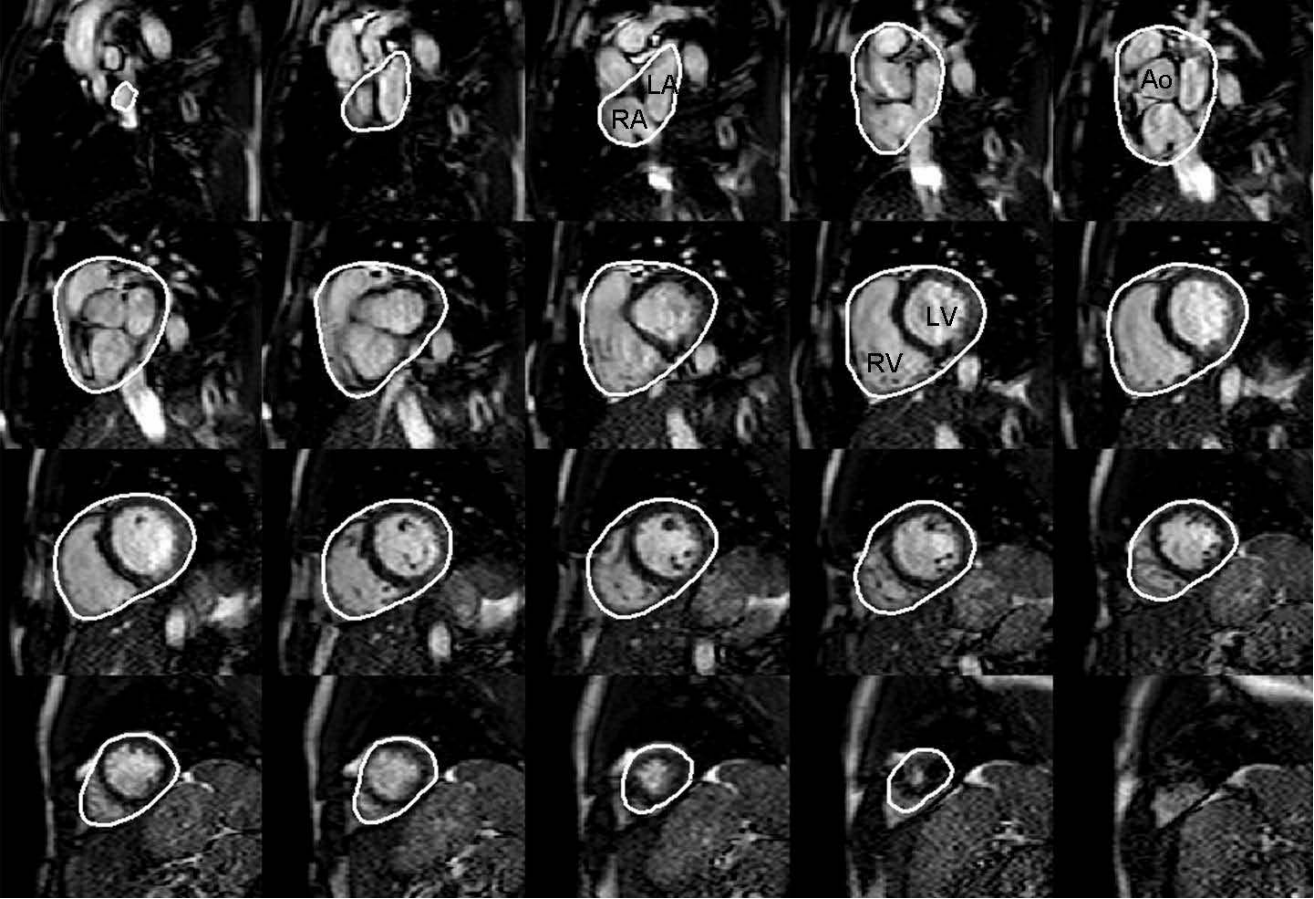
**Delineation of the pericardium enabling determination of the total heart volume from short-axis images**. The base is found in the upper left corner and apex in the lower right corner. The white line indicates the pericardium. The right atrium, left atrium, right ventricle and left ventricle are indicated. *Abbreviations: RA = right atrium, LA = left atrium, RV = Right ventricle, LV = left ventricle*.

Total heart volume was measured in the short-axis images by planimetry as previously described [16]. In short, THV was defined as the volume of all structures within the pericardium, including myocardium, blood pool, atria and pericardial fluid. This also includes the proximal parts of the great vessels covered by the pericardium. A region of interest was manually drawn around the pericardial border in all the short axis images of the heart in end-diastole (Figure. 1) using a freely available software (Segment 1.697; http://segment.heiberg.se) [17]. The summed area was then multiplied with slice thickness to obtain THV. This volumetric method used to determine THV has previously been used to study cardiac pumping mechanics [15,16,18,19]. A subset of subjects (11 athletes, 12 control subjects) was evaluated by a second observer for determination of inter-observer variability.

Left ventricular mass (LVM) and left ventricular end-diastolic volume (LVEDV) were measured in the short-axis images using planimetry, manually defining the endocardial and epicardial borders of the left ventricular myocardium. Papillary muscles were not included in LVM. Right ventricular end-diastolic volume (RVEDV) was also measured in short-axis images by manual delineation of the right ventricular endocardial border.

### Exercise test with gas analysis

Exercise testing was performed using an electronically braked cycle ergometer (Siemens Ergomed 940) and the gas analysis equipment Oxycon Champion (Jaeger, Hochberg, Germany). Serial VO_2 _values were obtained during the exercise test by calculating the average of all breaths taken during each 10-second period.

Athletes started at different workloads depending on their own preferences and need for a warm up period. Male athletes started at a work rate of 90, 110 or 130 Watts (W) and female athletes at 70, 90 or 110W. All test protocols for athletes then increased with 15W per 30s. The test protocol for the control group was based on age, weight and self-rated fitness level according to clinical praxis. Protocols were chosen to yield exercise duration of ~8-12 min [20]. The test continued until exhaustion or until the test subjects could not keep an even pace.  was defined as the highest value reached at the end of exercise.

A 12-lead ECG was acquired before, during and after exercise. Blood pressure was measured at rest in the supine position, every two minutes during exercise on the cycle sitting in an upright position, and in the supine position at rest after exercise. Measurements were done using a manual sphygmanometer. Resting heart rate was obtained from the ECG before the exercise test.

### Statistical analysis

Mann-Whitney non-parametric test was used to compare body habitus parameters, blood pressure, heart rate and  between groups, as well as cardiac dimensions such as THV, LVM, LVEDV, RVEDV, THV/BSA, LVM/THV, LVEDV/THV and RVEDV/THV. Univariable linear regression analysis was used to assess correlations between  and THV, LVM, LVEDV, RVEDV, body surface area (BSA) and height respectively. Linear regression was also performed to assess the relationship between LVEDV and RVEDV. Forward stepwise multivariable regression analysis, adjusted for gender, was performed to assess the independent predictive value of THV, LVM, LVEDV, RVEDV, BSA and height for determining . All statistical analysis was performed using SPSS 16.0 (Chicago, IL, USA). A p-value of less than 0.05 was considered statistically significant.

## Results

### Subject characteristics

Subject characteristics are presented in Tables 1 and 2. Male handball players trained endurance 3-5 hours/week and female handball players trained 2-3 hours/week. Male and female soccer players trained endurance for approximately 5-6 and 4-6 hours/week respectively. Male triathletes trained on average 11 hours/week (range 5.5-17.5) and had been training at this level for 6 years (range 3-10). Female triathletes trained on average 15 hours/week (range 13.5-17.5) and had been training for 5 years (range 3-7). Thirty-seven control subjects filled out the form on physical activity level. Eighty-four percent of them spent most of their day at work sitting. Ninety-five percent did some kind of physical activity at least 4 hours/week, such as bicycling or walking to work, and 57 percent added at least 2 hours/week of physical training such as team sports, running or weightlifting to their weekly physical activity.

**Table 1:** Subject characteristics

	Group	Number	Age (years)	Height (m)	Weight (kg)	BSA (m^2^)	Resting HR	Resting SBP	Resting DBP
**Males**	Control	n = 40	34 ± 10	1.81 ± 0.05	81 ± 10	2.0 ± 0.13	61 ± 9	129 ± 9	77 ± 7

	Handball	n = 11	25 ± 6**	1.86 ± 0.05**	87 ± 5*	2.1 ± 0.08**	56 ± 6	124 ± 8	70 ± 8**

	Soccer	n = 18	24 ± 5***	1.83 ± 0.05	79 ± 5	2.01 ± 0.08	59 ± 6	132 ± 5	75 ± 8

	Triathlon	n = 12	35 ± 9	1.84 ± 0.05*	81 ± 6	2.03 ± 0.10	56 ± 8	134 ± 8	75 ± 9

**Females**	Control	n = 20	36 ± 13	1.69 ± 0.06	66 ± 9	1.76 ± 0.12	62 ± 10	121 ± 9	70 ± 8

	Handball	n = 12	21 ± 2***	1.72 ± 0.03	68 ± 6	1.80 ± 0.08	60 ± 9	119 ± 5	70 ± 7

	Soccer	n = 12	23 ± 4***	1.70 ± 0.06	64 ± 6	1.75 ± 0.11	57 ± 8	119 ± 6	71 ± 6

	Triathlon	n = 6	31 ± 5	1.70 ± 0.06	62 ± 5	1.72 ± 0.11	51 ± 7*	121 ± 9	71 ± 9

* p < 0.05 **p < 0.01 ***p < 0.001 when compared to gender matched control subjects.

bpm = beats per minute, DBP = diastolic blood pressure, g = gram, kg = kilogram, m = meter, mmHg = millimetres mercury, ml/min = millilitres per minute, SBP = systolic blood pressure

**Table 2:** Total heart volume (THV), left ventricular mass (LVM), left and right end diastolic volume (LVEDV, RVEDV, ejection fraction (EF), stroke volume (SV), cardiac output(Q) and peak oxygen uptake () in males and females.

	Group	THV (ml)	LVM (g)	LVEDV (ml)	RVEDV (ml)	EF (%)	SV (ml)	Q (ml)	(ml/min)
**Males**	Control	853 ± 102 (706-1148)	118 ± 19 (90-165)	195 ± 28 (145-291)	211 ± 31 (144-302)	62 ± 7	120 ± 20	7.3 ± 1.4	3439 ± 520

	Handball	1050 ± 108 *** (928-1284)	146 ± 22 *** (119-184)	256 ± 41 *** (180-321)	256 ± 34 *** (198-311)	55 ± 4 **	141 ± 26 *	7.8 ± 1.3	4410 ± 422 ***

	Soccer	1040 ± 89 *** (901-1231)	147 ± 24 *** (91-189)	244 ± 28 *** (176-294)	255 ± 34 *** (160-299)	58 ± 6 *	142 ± 23 ***	8.3 ± 1.5 *	4122 ± 422 ***

	Triathlon	1104 ± 144 *** (914-1393)	167 ± 21 *** (129-198)	239 ± 26 *** (199-280)	254 ± 27 *** (202-290)	62 ± 6	147 ± 11 ***	8.2 ± 1.4	4765 ± 414 ***

**Females**	Control	673 ± 81 (544-816)	81 ± 15 (55-113)	160 ± 23 (116-195)	164 ± 26 (114-215)	62 ± 4	100 ± 16	6.2 ± 1.4	2429 ± 483

	Handball	757 ± 75 * (631-869)	95 ± 13 * (76-117)	188 ± 21 ** (150-216)	184 ± 22 * (155-235)	58 ± 4 **	108 ± 14	6.4 ± 1.1	3055 ± 250 ***

	Soccer	798 ± 86 ** (611-902)	95 ± 10 * (84-114)	194 ± 27 ** (145-233)	195 ± 26 ** (151-235)	55 ± 4 ***	106 ± 14	6.0 ± 1.3	3024 ± 242 **

	Triathlon	859 ± 85 ** (763-987)	115 ± 18 ** (99-138)	200 ± 28 ** (154-237)	220 ± 26 *** (197-270)	67 ± 4 *	133 ± 19 **	6.7 ± 1.0	3605 ± 117 ***

* p < 0.05 **p < 0.01 ***p < 0.001 when compared to gender matched control subjects. g = gram, min = minute, ml = millilitre

### Peak oxygen uptake in relation to cardiac variables and body habitus

**Figure 2:**
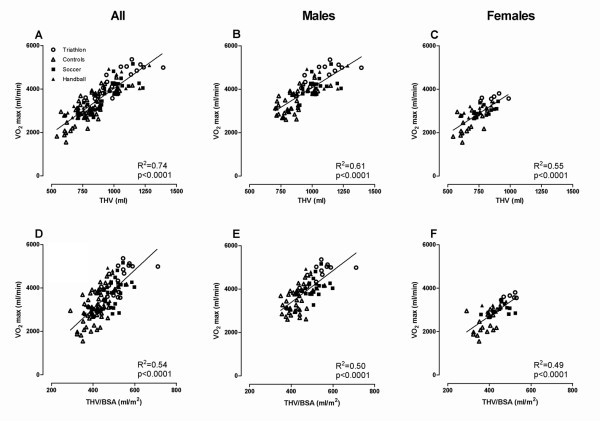
**The relationship between total heart volume (THV) and peak oxygen uptake ()**. The upper panel shows the relationship between absolute THV and  for A) all subjects, B) males and C) females. The lower panel shows the relationship between THV normalized for body surface area (BSA) and  for D) all subjects, E) males and F) females. For both males and females,  was significantly correlated to THV and to THV/BSA. *Abbreviations: BSA = body surface area, THV = total heart volume*,  = *peak oxygen uptake*

For both males and females,  was significantly correlated to THV (Figure. 2A-C) and THV normalized for BSA (Figure. 2D-F). Furthermore,  was significantly correlated to LVM, LVEDV, RVEDV, BSA and height (Table 3). By multivariable analysis, THV and LVM were shown to be independent predictors of  (R^2 ^= 0.74, p < 0.001 for THV alone, R^2 ^= 0.78, p < 0.001 when including LVM) (Table 3), independent of gender. When the study population was divided by gender, there was a significant correlation between  and all variables studied, except for BSA and height in females.

**Table 3:** Univariable linear regression for total heart volume (THV), left ventricular mass (LVM), body surface area (BSA), left ventricular end-diastolic volume (LVEDV), right ventricular end-diastolic volume (RVEDV) and height in male subjects, female subjects and all subjects respectively.

	Univariable regression Males		Univariable regression Females		Univariable regression All subjects		Multivariable regression All subjects	
	**R^2^**	**p**	**R^2^**	**p**	**R^2^**	**p**	**β**	**p**

THV (ml)	0.61	<0.0001	0.55	<0.0001	0.74	<0.0001	0.512	<0.0001

LVM (g)	0.52	<0.0001	0.64	<0.0001	0.71	<0.0001	0.398	<0.0001

LVEDV (ml)	0.42	<0.0001	0.39	<0.0001	0.55	<0.0001	na	na

RVEDV (ml)	0.41	<0.0001	0.45	<0.0001	0.60	<0.0001	na	na

BSA (m^2^)	0.14	<0.001	0.02	ns	0.40	<0.0001	na	na

Height (m)	0.14	<0.001	0.04	ns	0.40	<0.0001	na	na

g = gram, m = meter, ml = milliliter

Multivariable linear regression analysis for all subjects in relation to .

The inter-observer variability for determination of THV was -5 ± 37 ml (R^2 ^= 0.96, p < 0.001).

### Left and right ventricular dimensions

**Figure 3:**
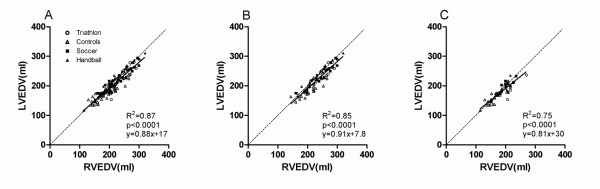
**The relationship between the left ventricular end-diastolic volume (LVEDV) and right ventricular end-diastolic volume (RVEDV) for: A) all subjects, B) males and C) females**. The solid line is the regression line and the dashed line is the line of identity. There is a balanced enlargement of the left and right ventricle with increased amount of endurance training. *Abbreviations: LVEDV = left ventricular end-diastolic volume, RVEDV = right ventricular end-diastolic volume*.

Figure 3 shows the relationship between LVEDV and RVEDV. As the LVEDV increased, the RVEDV increased in the same order of magnitude (R^2 ^= 0.87, p < 0.001).

### Gender aspects

**Figure 4:**
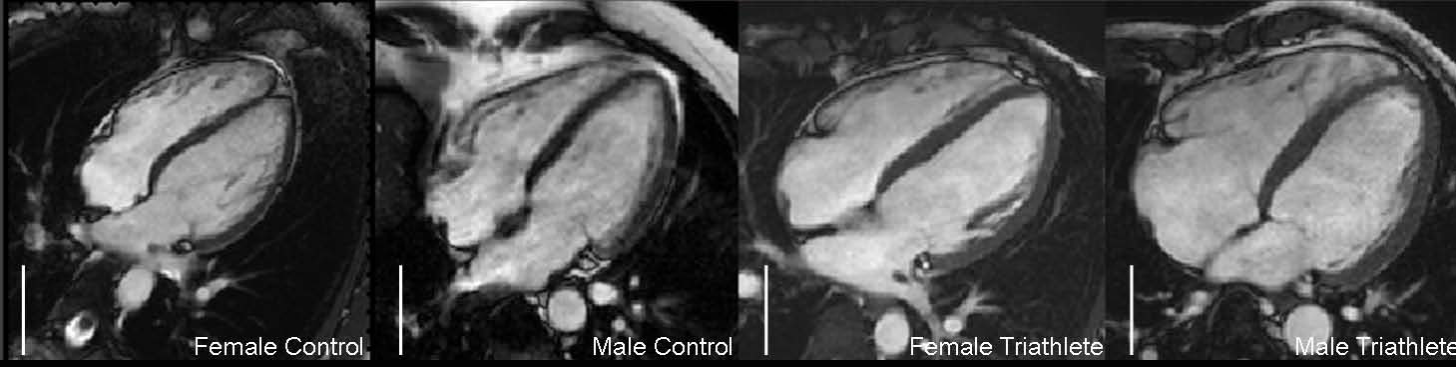
**Long axis images of the heart in a female control, male control, female triathlete and male triathlete**. This figure illustrates the increase in cardiac dimensions with endurance training. Note that the female triathlete (height 1.80 m, weight 70 kg) has a larger heart than the male control subject (1.81 m, 80 kg).

**Figure 5:**
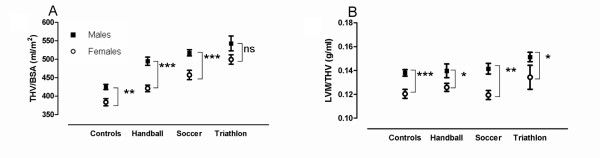
**Comparison of cardiac dimensions between males and females of the same sport: A) total heart volume normalized for body surface area (THV/BSA), B) left ventricular mass normalized for total heart volume (LVM/THV)**. Error bars denotes standard error of the mean. All male subject groups had a significantly higher THV/BSA and LVM/THV when compared to females, except for THV/BSA in triathletes. *Abbreviations: C = control subjects, H = handball players, LVM = left ventricular mass, S = soccer players, T = triathletes, THV = total heart volume*

Male control subjects, handball players, soccer players and triathletes all had significantly higher THV, LVM, LVEDV and RVEDV when compared to sport-matched females. Figure 4 illustrates the difference between males and females as well as controls compared to triathletes. The stroke volume (SV) was significantly higher in male controls, handball players and soccer players, but not in male triathletes (p = 0.092 for triathletes). The cardiac output (), however, was significantly higher in all male groups (controls p = 0.033, handball p = 0.012, soccer p < 0.001, triathlon p = 0.025). As seen in Figure 5A and 5B, all male subject groups had a significantly higher THV/BSA and LVM/THV when compared to females, except for THV/BSA in triathletes. LVEDV/THV did not differ between males and females for any of the groups and RVEDV/THV was significantly different only between male and female triathletes (p = 0.049).

## Discussion

To our knowledge, the present study is the first to show that THV is a strong independent predictor of  in both males and females. Furthermore, this study shows that the cardiac dimensions increase in the same order of magnitude in both males and females, and the athlete's heart is significantly larger than a normal heart with a balanced enlargement of the left and right ventricles.

The significant correlation between THV and  found when including all study subjects remained statistically significant when the study population was divided by gender. In a previous study by Ekblom et al. [21], the relationship between  and THV determined from chest x-ray was studied in a small group of 13 male endurance athletes, which gave a limited range of the variables studied. Therefore, the physiological relationship between  and THV was not revealed. To overcome this limitation, the present study, by design, includes a large study population with a continuous increased level of long term endurance training in order to show the physiological relationship between  and THV. The findings of the present study show a strong correlation between  and THV, even when normalizing for BSA. Hence, for a given BSA, an increased THV is predictive of a higher .

 cannot be fully explained by central factors, such as THV. It is also dependent on peripheral factors such as haemoglobin concentration and the atriovenous oxygen difference, as described by the Fick principle. However, previous studies have shown that although the peripheral factors explain part of the variability in the relationship between  and [21,22], the major limiting factor for oxygen delivery is the cardiorespiratory system [23].

Cardiac performance is commonly assessed by determining cardiac output (), which has been shown to be associated with [23]. Maximum  is dependent on maximal heart rate and SV and has been shown to increase by long term endurance training [21]. Furthermore, it has previously been shown that the maximal heart rate does not increase by long term endurance training [8]. Thus, an increased maximal  induced by endurance training would be explained by an increased SV, due to either an increase in ventricular dimensions or improved pumping mechanics. The data in the present study suggests that an increase in cardiac dimensions may explain parts of an expected increase in maximal  leading to an increased . To which extent exercise induced changes in pumping mechanics contributes to the increased cardiac performance is not yet fully understood. Previous studies using echocardiography have shown conflicting results of training effects on left ventricular filling, with both improved diastolic filling in athletes as well as no differences between athletes and controls (for review, see George et al. [24]). With new promising techniques, such as velocity encoded CMR, cardiac pumping in athletes can be further elucidated.

### Gender Aspects

In the present study we show that the relationship between THV and  is gender independent. Furthermore, both the absolute THV and THV normalized for BSA increase in the same order of magnitude in both males and females. In line with the work by Petersen et al. [25], males have larger ventricular dimensions when compared to females and both genders show a physiologically enlarged heart due to long term endurance training.

Total heart volume normalized for BSA was significantly higher in males compared to females, except for triathletes. For the soccer players and handball players, the male athletes had more endurance training than female athletes. For the triathletes, however, the female athletes had an average of 4 hours/week more endurance training than the male athletes, indicating the importance of endurance training on THV.

Left ventricular mass normalized for THV was significantly higher in all male groups compared to females. It has previously been shown that males have a higher systolic blood pressure during exercise, which may stimulate left ventricular hypertrophy due to increased afterload [26,27]. Furthermore, the higher level of testosterone in males compared to females may also partly explain the differences in LVM. Estrogens have been shown to have the opposite effect to testosterone on cardiac remodelling [28,29].

The relationship between the LVEDV and RVEDV was similar for males and females (Figure. 3B and 3C) with a slightly larger RVEDV compared to LVEDV. Although there was a small difference in the slope of the regression lines between males and females, the data in the present study suggests that the athlete's heart is physiologically enlarged with a balance between the left and right ventricles in both genders, as has previously been shown [7,25,30].

The physiology and potential pathophysiology of the athlete's heart has been discussed in several studies [31-33]. It is a clinical challenge to determine if a heart is physiologically or pathologically enlarged. In the presence of cardiac pathology, THV may not be proportional to , thus indicating an abnormal enlargement of the heart. In addition, cardiac pathology may cause an imbalance of the enlargement of the left and right ventricle, enabling discrimination between a physiological or pathological enlarged heart.

### Limitations

The findings in the present study should be interpreted in the light of some limitations. First, the study population consists of heterogeneous groups of study subjects which may be considered a limitation. However, in order to study the physiological relationship between  and THV, a continuous wide range of endurance training is needed. Therefore, the study, by design, includes both males and females with varying degree of long term endurance training. Second, the healthy control subjects were recruited via an advertisement and there might be a possible selection bias. The control population may have been better trained than the general population, since well trained controls may be more likely to volunteer in a study like the present study. This might explain why the male and female triathletes were the only groups with a resting HR significantly lower than the controls. Third, it is known that test subjects may have problems reaching their true  on an ergometer cycle and  may be underestimated by up to ten percent, which has to be considered when interpreting the results. Finally, the study setup did not allow blood sampling, and it has previously been shown that haemoglobin concentration affects .

## Conclusion

This study has shown that THV is a strong, independent predictor of maximal work capacity for both males and females. Long term endurance training is associated with a physiologically enlarged heart with a balance between the left and right ventricular dimensions in males and females.

## Competing interests

The authors declare that they have no competing interests.

## Authors' contributions

**KS **made substantial contribution to the conception and design of the study, the acquisition of data and data analysis, drafted the manuscript and has given full approval of the version to be published. **HE **contributed to the conception and design of the study, the data analysis and has critically revised the manuscript and approved the final version. **TB **contributed to the conception and design of the study, the acquisition of data and has critically revised the manuscript and approved the final version. **MC **contributed to the conception and design of the study, the data analysis and has critically revised the manuscript and approved the final version. **HM **made substantial contribution the acquisition of data and has critically revised the manuscript and approved the final version. **BW **made substantial contribution to the conception and design of the study, the data analysis and has critically revised the manuscript and approved the final version. **HA **made substantial contribution to the conception and design of the study, and has critically revised the manuscript and approved the final version.
